# Classifier-Derived Guidance for Diffusion-Based Medical Dataset Distillation

**DOI:** 10.3390/bioengineering13101100

**Published:** 2026-09-23

**Authors:** Shuailin Du, Guang Li, Ren Togo, Takahiro Ogawa, Miki Haseyama

**Affiliations:** 1 Graduate School of Information Science and Technology, Hokkaido University, N-14, W-9, Kita-ku, Sapporo 060-0814, Hokkaido, Japan; du@lmd.ist.hokudai.ac.jp; 2 Education and Research Center for Mathematical and Data Science, Hokkaido University, N-13, W-10, Kita-ku, Sapporo 060-0813, Hokkaido, Japan; guang@lmd.ist.hokudai.ac.jp; 3 Faculty of Information Science and Technology, Hokkaido University, N-14, W-9, Kita-ku, Sapporo 060-0814, Hokkaido, Japan; togo@lmd.ist.hokudai.ac.jp (R.T.); ogawa@lmd.ist.hokudai.ac.jp (T.O.)

**Keywords:** dataset distillation, medical imaging, diffusion models, classifier guidance, synthetic data

## Abstract

Medical image analysis often relies on large annotated datasets, whose collection, storage, and sharing are costly and constrained by privacy concerns. Dataset distillation offers a potential solution by constructing compact synthetic datasets that retain task-relevant information from the original data. However, generative distillation methods may not adequately preserve class-specific information in medical images, where clinically distinct classes often exhibit similar visual characteristics. To address this issue, we propose a classifier-derived guidance framework for diffusion-based medical dataset distillation. The framework uses predictions from a pretrained noise-conditioned classifier to guide diffusion sampling through three objectives. Classifier guidance (CG) promotes class alignment, whereas entropy and margin guidance encourage boundary-oriented sampling. We analyze guidance-strength sensitivity on three medical imaging datasets across images-per-class (IPC) budgets and evaluate validation-selected COVID-19 Radiography configurations on the test subset using three downstream architectures. Classifier diagnostics distinguish prediction–conditioning agreement from downstream utility, while learning curves illustrate differences in validation behavior despite low training loss. These findings characterize the configuration-dependent utility of classifier-derived guidance for compact synthetic medical datasets.

## 1. Introduction

Deep learning has advanced medical image analysis across a range of imaging modalities and clinical tasks, including disease detection, tumor classification, and cell segmentation [1,2]. However, the performance of modern AI models remains strongly dependent on the scale, diversity, and quality of their training data. Access to suitable medical datasets is often limited by restricted data availability and barriers to data sharing [3,4]. Privacy concerns, heterogeneous data formats, legal requirements, and institutional constraints further complicate the collection and exchange of the large and diverse datasets required for robust model development [5].

In this setting, dataset distillation (DD) provides a data-centric approach to medical data sharing. As illustrated in Figure 1, rather than transferring the complete source dataset, DD constructs a compact synthetic dataset that retains task-relevant information for downstream model training [6,7]. Existing DD methods can be broadly divided into generative and non-generative approaches. Non-generative methods commonly optimize synthetic samples by matching the gradients, training trajectories, or feature distributions induced by the original data [8,9]. Despite their effectiveness, the repeated optimization of synthetic samples through model training or feature extraction can be computationally demanding and difficult to extend to high-resolution data. These limitations are particularly relevant to medical imaging, where image collections may be large and diagnostically important structures can be fine-grained [10].

Generative DD offers an alternative by using learned generative priors to produce compact synthetic training sets. Existing studies have investigated both GAN-based and diffusion-based formulations [11,12]. Recent diffusion-based DD methods have focused on improving the representativeness, diversity, and sampling efficiency of distilled data [13,14]. For example, Minimax Diffusion introduces a minimax objective to improve the coverage and diversity of distilled samples on challenging benchmarks [14]. Related advances in diffusion-model acceleration have also used trajectory distribution matching to support efficient few-step generation while retaining the flexibility of multi-step sampling [15]. Together, these developments indicate that diffusion models provide a flexible generative foundation for constructing compact training datasets. Within this generative setting, the present study focuses on how classifier-derived sampling objectives affect the downstream training utility of compact medical datasets. Specifically, we compare guidance that strengthens target-class alignment with guidance that encourages exploration of uncertain regions near classifier decision boundaries.

Most generative DD methods, however, have been evaluated primarily on natural-image benchmarks, leaving their effectiveness on high-resolution and structurally complex medical images insufficiently understood [16]. Medical-image classes may be distinguished by subtle variations in texture, density, morphology, or localized abnormalities, which can make inter-class discrimination particularly difficult [17]. These characteristics may require medical DD methods to preserve fine-grained, localized, and class-discriminative structures that are less prominent in conventional natural-image benchmarks. If such information is not adequately retained, a compact synthetic dataset may fail to support reliable class separation. Preserving discriminative information while maintaining useful intra-class variation is therefore a central challenge in medical dataset distillation [18].

Motivated by this challenge, we investigate classifier-derived guidance for diffusion-based medical dataset distillation. Inspired by measurement-guided diffusion for medical image synthesis [19], the proposed framework uses a separately trained noise-conditioned classifier to modify the reverse diffusion trajectory. We formulate three classifier-derived guidance objectives with complementary roles. Classifier guidance promotes alignment with the target class, whereas entropy guidance (EG) and margin guidance (MG) encourage boundary-oriented sampling intended to produce informative hard examples. We systematically evaluate these objectives across three medical imaging datasets, multiple images-per-class (IPC) budgets, guidance strengths, and downstream architectures.

The main contributions of this study are summarized as follows:

- We adapt class-alignment and boundary-oriented classifier guidance to diffusion-based medical dataset distillation.
- We systematically characterize the effects of three guidance objectives across medical imaging modalities, IPC budgets, and guidance strengths.
- We examine both beneficial operating regimes and failure modes of classifier-derived guidance through downstream evaluation, generated-sample analysis, and feature-space projections.

## 2. Related Work

### 2.1. Dataset Distillation

DD aims to construct a compact synthetic dataset that preserves the training utility of a substantially larger source dataset [6]. Unlike knowledge distillation, which transfers predictive information from a teacher model to a student model, DD compresses information at the data level. Early optimization-based DD methods focused on matching the training signals induced by synthetic and real data. Gradient-matching methods optimize synthetic samples so that they produce gradients similar to those obtained from real data [8,9]. Subsequent studies have improved synthetic-data optimization through efficient parameterization, accumulated trajectory matching, and loss-curvature information [20,21,22,23]. These methods seek to preserve how real data influence the optimization process rather than matching only an isolated training signal.

Distribution-matching methods provide another major direction by aligning the feature statistics of synthetic and real data in selected embedding spaces [24,25]. For example, DANCE aligns real and synthetic distributions through complementary feature views, allowing the distilled dataset to capture information represented at different feature levels [26]. Compared with gradient- and trajectory-based formulations, distribution matching generally avoids explicitly reproducing long model-training processes during synthetic-data optimization.

More recently, generative DD methods have introduced learned generative priors into the construction of distilled datasets [14,27]. Rather than optimizing every synthetic image independently, these methods use a generative model to capture the source-data distribution and produce compact training samples. Earlier approaches adopted GAN-based and VAE-based formulations [28,29], whereas more recent studies have increasingly employed diffusion models [13,30,31]. Diffusion-based DD methods guide the reverse sampling process using objectives designed to improve the representativeness, informativeness, or diversity of the generated samples. This formulation provides a flexible mechanism for controlling sample generation and has shown promising performance on natural-image benchmarks.

### 2.2. Dataset Distillation in Medical Imaging

Dataset distillation has also been investigated for medical image analysis and data sharing. Soft-label gastric X-ray image distillation constructs compact synthetic representations that transfer task-relevant information without directly distributing the complete source dataset [10]. Subsequent work extends this formulation to compressed gastric image generation and studies how distilled data can support model training between data contributors and recipients [16,32]. More recent studies have evaluated the feasibility of DD across different medical imaging tasks and datasets [18]. High-order progressive trajectory matching further adapts training-dynamics matching to medical image dataset distillation by incorporating higher-order information from model-training trajectories [33].

Generative approaches provide another direction for constructing compact and shareable medical datasets. MedSynth, for example, uses a generative model to encode information from a source medical dataset and generate representative samples for downstream model training [34]. Together with compressed medical image generation [32], these studies demonstrate the potential of generative models for reducing the amount of medical data that must be directly transferred.

Despite this progress, generative DD for medical imaging remains less extensively studied than its natural-image counterpart. In particular, the effects of classifier-derived guidance during diffusion sampling have not been systematically investigated in medical dataset distillation. It remains unclear how guidance objectives that promote target-class alignment or decision-boundary exploration affect downstream utility across medical imaging modalities, IPC budgets, and guidance strengths. The present study addresses this gap by investigating class-alignment and boundary-oriented guidance within a diffusion-based medical DD framework.

## 3. Preliminaries: Diffusion Models

Diffusion models generate samples by learning to reverse a progressive noising process. Denoising Diffusion Probabilistic Models (DDPMs) [35] define a forward process that gradually transforms a clean sample $x_{0}∼q_{\mathrm{data}}\left(x\right)$ into Gaussian noise through a sequence of latent variables ${\left\{x_{t}\right\}}_{t=0}^{T}$, where $x_{t}\in R^{d}$. Let $\alpha_{t}=1-\beta_{t}$ and ${\bar{\alpha }}_{t}=\prod_{s=1}^{t}\alpha_{s}$, where $\beta_{t}$ denotes the variance schedule. A noisy observation at timestep *t* can then be sampled directly as

(1) $$x_{t}=\sqrt{{\bar{\alpha }}_{t}}x_{0}+\sqrt{1-{\bar{\alpha }}_{t}}\epsilon ,\;\epsilon ∼N\left(0,I\right).$$

The reverse process starts from $x_{T}∼N\left(0,I\right)$ and progressively recovers a sample from the data distribution. A noise-prediction network $\epsilon_{\theta }\left(x_{t},t\right)$ estimates the noise component contained in $x_{t}$. Based on Equation (1), the corresponding prediction of the clean sample is

(2) $$f_{\theta }\left(x_{t},t\right):=\frac{x_{t}-\sqrt{1-{\bar{\alpha }}_{t}}\epsilon_{\theta }\left(x_{t},t\right)}{\sqrt{{\bar{\alpha }}_{t}}}.$$

The family of reverse processes associated with DDIM sampling [12] can be expressed through the conditional distribution

(3) $$q_{\sigma }\left(x_{t-1}|x_{t},x_{0}\right)=N\left(\sqrt{{\bar{\alpha }}_{t-1}}x_{0}+\sqrt{1-{\bar{\alpha }}_{t-1}-\sigma_{t}^{2}}\frac{x_{t}-\sqrt{{\bar{\alpha }}_{t}}x_{0}}{\sqrt{1-{\bar{\alpha }}_{t}}};\sigma_{t}^{2}I\right),$$

where $\sigma_{t}$ controls the stochasticity of the reverse process. Replacing the unknown $x_{0}$ with its prediction $f_{\theta }\left(x_{t},t\right)$ gives the following reverse update:

(4) $$\begin{array}{cc} x_{t-1}= & \underset{\mathrm{predicted}\;clean\mathrm{-s}ample\;\mathrm{component}}{\underset{︸}{\sqrt{{\bar{\alpha }}_{t-1}}f_{\theta }\left(x_{t},t\right)}}\\ \; & +\underset{\mathrm{direction}\;\mathrm{toward}\;x_{t}}{\underset{︸}{\sqrt{1-{\bar{\alpha }}_{t-1}-\sigma_{t}^{2}}\;\epsilon_{\theta }\left(x_{t},t\right)}}+\underset{\mathrm{stochastic}\;\mathrm{component}}{\underset{︸}{\sigma_{t}z_{t}}},\;z_{t}∼N\left(0,I\right). \end{array}$$

Different choices of $\sigma_{t}$ define different sampling processes while using the same noise-prediction network. When $\sigma_{t}^{2}$ matches the posterior variance of the forward process, the update recovers DDPM sampling. When $\sigma_{t}=0$ for all *t*, the stochastic component in Equation (4) vanishes, and the reverse trajectory becomes deterministic for a fixed initial noise $x_{T}$. This special case corresponds to the Denoising Diffusion Implicit Model (DDIM) [12], which can be trained using the standard DDPM objective.

Equation (4) shows that the predicted noise $\epsilon_{\theta }\left(x_{t},t\right)$ directly determines the reverse trajectory. Consequently, replacing it with a classifier-guided noise estimate can steer the generated sample toward features associated with a target class $y_{i}$. The next section introduces the classifier-derived objectives used to construct this guided noise estimate.

## 4. Methodology

As illustrated in Figure 2, the proposed framework incorporates classifier-derived guidance into class-conditional DDIM sampling for medical dataset distillation. The guidance objectives serve two complementary roles: promoting alignment with the target class and encouraging exploration near classifier decision boundaries. The framework consists of four components: model pretraining, classifier-derived guidance objectives, guided DDIM sampling, and conditioning-label assignment.

### 4.1. Pretraining

Let $D_{\mathrm{train}}={\left\{\left(x_{0}^{\left(i\right)},y^{\left(i\right)}\right)\right\}}_{i=1}^{N}$ denote the source training dataset, where $x_{0}^{\left(i\right)}$ and $y^{\left(i\right)}$ represent the *i*-th training image and its class label, respectively. We first train a class-conditional diffusion model on $D_{\mathrm{train}}$. At diffusion timestep *t*, a noisy observation $x_{t}$ is constructed according to Equation (1). The noise-prediction network $\epsilon_{\theta }\left(x_{t},t,y\right)$ receives the noisy image, diffusion timestep, and class condition, and is optimized using

(5) $$L_{\mathrm{diff}}\left(\theta \right)=E_{(x_{0},y),t,\epsilon }\left[{∥\epsilon -\epsilon_{\theta }\left(x_{t},t,y\right)∥}_{2}^{2}\right].$$

This objective trains the network to estimate the noise component at different diffusion timesteps while retaining information associated with the class condition.

We independently train a noise-conditioned guidance classifier $p_{\phi }\left(y|x_{t},t\right)$ on the same source training split. The classifier adopts the downsampling path and attention-pooling components of a U-Net-based architecture. It receives the noisy observation $x_{t}$ and the corresponding timestep embedding and is optimized using

(6) $$L_{\mathrm{cls}}\left(\phi \right)=E_{(x_{0},y),t,\epsilon }\left[-\log p_{\phi }\left(y|x_{t},t\right)\right].$$

The diffusion model and guidance classifier are trained independently. The guidance classifier is used only during reverse sampling and does not modify the training objective of the diffusion model. The guidance classifier is distinct from the downstream classifiers used to evaluate the compact datasets. During sampling, its parameters remain fixed, and gradients are computed with respect to the current noisy image rather than the classifier parameters. Independent pretraining does not itself prevent classifier errors or biases from influencing generation. We assess the classifier’s accuracy on clean real validation images and present an illustrative analysis of prediction–conditioning agreement for generated images in Section 5.2.

### 4.2. Classifier-Derived Guidance Objectives

We consider three classifier-derived guidance objectives with two functional roles. Classifier guidance (CG) promotes alignment with a specified target class, whereas entropy guidance (EG) and margin guidance (MG) encourage boundary-oriented sampling. Each objective is applied separately during the reverse process.

For a target class label $y_{i}\in \left\{0,\ldots ,C-1\right\}$, the CG objective is defined as

(7) $$M_{\mathrm{CG}}\left(x_{t},t;y_{i}\right)=\log p_{\phi }\left(y_{i}|x_{t},t\right).$$

Maximizing this objective increases the probability assigned to the target class and guides the reverse trajectory toward features that the guidance classifier associates with $y_{i}$. The EG objective is defined as

(8) $$M_{\mathrm{EG}}\left(x_{t},t\right)=-\sum_{c=0}^{C-1}p_{\phi }\left(c|x_{t},t\right)\log p_{\phi }\left(c|x_{t},t\right),$$

where *C* denotes the number of classes. Maximizing this objective increases the predictive entropy of the guidance classifier and encourages sampling in regions of high class uncertainty, which may lie near decision boundaries.

The MG objective is defined as

(9) $$M_{\mathrm{MG}}\left(x_{t},t\right)=-\left[p_{\phi }\left({\hat{y}}_{1}|x_{t},t\right)-p_{\phi }\left({\hat{y}}_{2}|x_{t},t\right)\right],$$

where

(10) $${\hat{y}}_{1}=\underset{c}{\arg\max}\;p_{\phi }\left(c|x_{t},t\right),\;{\hat{y}}_{2}=\underset{c\neq {\hat{y}}_{1}}{\arg\max}\;p_{\phi }\left(c|x_{t},t\right)$$

denote the classes with the highest and second-highest predicted probabilities, respectively. At each reverse timestep, these two classes are determined from the current classifier prediction before the gradient with respect to $x_{t}$ is computed. Maximizing $M_{\mathrm{MG}}$ reduces the probability gap between the two leading classes and encourages the trajectory to approach the corresponding decision boundary. Unlike EG, which considers uncertainty over the complete class distribution, MG focuses on the two most probable classes.

### 4.3. Guided DDIM Sampling

Let $M_{m}$ denote the selected guidance objective, where m∈{CG,EG,MG}. At each reverse diffusion timestep, the guidance gradient is computed as

(11) $$g_{t}=\nabla_{x_{t}}M_{m},\;m\in \left\{\mathrm{CG},\mathrm{EG},\mathrm{MG}\right\}.$$

For all three objectives, the class-conditional diffusion model remains conditioned on $y_{i}$ throughout the reverse process. The guidance gradient modifies the diffusion model’s noise prediction as follows:

(12) $${\hat{\epsilon }}_{\theta }\left(x_{t},t,y_{i}\right)=\epsilon_{\theta }\left(x_{t},t,y_{i}\right)-s\sqrt{1-{\bar{\alpha }}_{t}}\;g_{t},$$

where *s* denotes the guidance strength. Setting s=0 recovers the unguided class-conditional DDIM baseline. The guided prediction of the clean sample is then calculated as

(13) $${\hat{x}}_{0}=\frac{x_{t}-\sqrt{1-{\bar{\alpha }}_{t}}{\hat{\epsilon }}_{\theta }\left(x_{t},t,y_{i}\right)}{\sqrt{{\bar{\alpha }}_{t}}}.$$

Let $\tau_{1}<\cdots <\tau_{K}$ denote the retained diffusion timesteps, with $\tau_{0}=0$ and ${\bar{\alpha }}_{\tau_{0}}=1$. For deterministic DDIM sampling, the update from $\tau_{j}$ to $\tau_{j-1}$ is

(14) $$x_{\tau_{j-1}}=\sqrt{{\bar{\alpha }}_{\tau_{j-1}}}\;{\hat{x}}_{0}+\sqrt{1-{\bar{\alpha }}_{\tau_{j-1}}}\;{\hat{\epsilon }}_{\theta }\left(x_{\tau_{j}},\tau_{j},y_{i}\right).$$

Classifier-derived guidance modifies only the reverse sampling trajectory and therefore does not require retraining the diffusion model. The same pretrained generator can consequently be used to evaluate different guidance objectives and strengths.

### 4.4. Label Assignment for Generated Samples

Each generated image requires a label for downstream training. We distinguish the conditioning label used during generation from the label subsequently assigned to the generated image. A predefined class label $y_{i}$ is supplied to the class-conditional diffusion model throughout sampling. This label specifies the intended class; it is not inherited from a particular source image, because sampling starts from Gaussian noise.

For CG, the guidance objective also promotes the same target class. EG and MG instead encourage predictive uncertainty while retaining the diffusion model’s class condition. We retain $y_{i}$ as the downstream training label to preserve this class-conditioned, boundary-oriented design. Replacing it with the guidance classifier’s top-1 prediction would make the assigned label directly dependent on the classifier being used to steer generation.

However, conditioning on $y_{i}$ does not guarantee that the final image preserves the intended class semantics. Retaining conditioning labels can therefore introduce label noise when generation departs from the intended class. Prediction–conditioning agreement is reported in Section 5.2 to examine the behavior of this labeling strategy. The complete guided sampling procedure is summarized in Algorithm 1.

We use T=1000 training noise steps and K=250 deterministic DDIM updates (η=0), with timestep respacing set to ddim250. In the notation of Section 3, $\tau_{j}=1+4\left(j-1\right)$ for j=1,…,250, corresponding to the zero-based implementation timesteps 0,4,…,996, which are visited in reverse order.

Algorithm 1 generates one labeled image. At each reverse timestep, the guidance classifier first provides the class-probability distribution. The selected guidance objective is evaluated using its definition in Equations (7)–(9), and its gradient modifies the noise prediction through Equation (12). CG increases the target-class probability, EG increases predictive entropy, and MG reduces the probability gap between the two leading classes. These objectives are applied separately, not combined within a sampling trajectory.

To construct a compact dataset with *k* images per class, the procedure is repeated *k* times for each of the *C* class conditions, producing Ck image–label pairs. A separate downstream classifier is subsequently trained on this compact dataset. The pretrained diffusion model and guidance classifier are not updated during this downstream training.
**Algorithm 1** Classifier-derived guided DDIM sampling.  1:**Input:** Class-conditional diffusion model $\epsilon_{\theta }\left(x_{t},t,y\right)$  2:**Input:** Pretrained guidance classifier $p_{\phi }\left(y|x_{t},t\right)$  3:**Input:** Class condition $y_{i}$  4:**Input:** Retained timesteps $\tau_{1}<\cdots <\tau_{K}$  5:**Input:** Guidance objective m∈{CG,EG,MG}  6:**Input:** Guidance strength *s*  7:**Initialize:** $x_{\tau_{K}}∼N\left(0,I\right)$  8:**for** j=K,…,1**do**  9:      $t\leftarrow \tau_{j}$10:      $g_{t}\leftarrow \nabla_{x_{t}}M_{m}$11:      $\epsilon_{t}\leftarrow \epsilon_{\theta }\left(x_{t},t,y_{i}\right)$12:      ${\hat{\epsilon }}_{t}\leftarrow \epsilon_{t}-s\sqrt{1-{\bar{\alpha }}_{t}}\;g_{t}$13:      ${\hat{x}}_{0}\leftarrow \frac{x_{t}-\sqrt{1-{\bar{\alpha }}_{t}}\;{\hat{\epsilon }}_{t}}{\sqrt{{\bar{\alpha }}_{t}}}$14:      $x_{\tau_{j-1}}\leftarrow \sqrt{{\bar{\alpha }}_{\tau_{j-1}}}\;{\hat{x}}_{0}+\sqrt{1-{\bar{\alpha }}_{\tau_{j-1}}}\;{\hat{\epsilon }}_{t}$15:**end for**16:**Output:** $\left(x_{0},y_{i}\right)$

### 4.5. Privacy Scope and Unaddressed Risks

The sharing workflow releases compact synthetic datasets rather than source images. Both pretrained models are learned from the source data, and the framework does not include differential privacy. Privacy assessment should examine source-image memorization and training-membership disclosure under the intended release setting, distinguishing access to synthetic images from access to model parameters. Differentially private training is a possible extension that would require privacy accounting for the data-dependent components of the pipeline.

## 5. Experiments

We analyze guidance-strength sensitivity on three medical imaging datasets using ResNet-18. We further evaluate the validation-selected COVID-19 Radiography configurations on the test subset using ResNet-18, ConvNet6, and ResNetAP-10. Table 1 summarizes the three datasets and the main experimental settings used in this study.

### 5.1. Experimental Datasets and Settings

Datasets.

OCTMNIST [36,37] contains retinal optical coherence tomography images collected from 4686 adult patients at multiple medical centers, including the Shiley Eye Institute, the California Retinal Research Foundation, the Medical Center Ophthalmology Associates, Shanghai First People’s Hospital, and Beijing Tongren Eye Center. The dataset comprises 109,309 images from four diagnostic classes: 37,455 images of choroidal neovascularization, 11,598 images of diabetic macular edema, 8866 images of drusen, and 51,390 normal images. We retain the official training split of 97,477 images, including 33,484, 10,213, 7754, and 46,026 images from the four respective classes. The official validation and test splits contain 10,832 and 1000 images, respectively. These subsets are kept separate in the revised evaluation protocol.

BloodMNIST [36,38] contains microscopic images of peripheral blood cells collected from healthy individuals at the Core Laboratory of the Hospital Clinic of Barcelona. Expert clinical pathologists annotated 17,092 images from eight cell classes: 3329 neutrophils, 3117 eosinophils, 1218 basophils, 1214 lymphocytes, 1420 monocytes, 2895 immature granulocytes, 1551 erythroblasts, and 2348 platelets. We use the official training, validation, and test splits, containing 11,959, 1712, and 3421 images, respectively.

The COVID-19 Radiography dataset [39,40] contains chest X-ray images compiled by researchers and medical professionals from Qatar University and the University of Dhaka. It comprises 21,165 images from four classes: 3616 COVID-19-positive cases, 10,192 normal cases, 6012 cases with lung opacity, and 1345 cases with viral pneumonia. We retain the original training split of 16,933 images for diffusion-model and guidance-classifier training and Random Real subset construction. The remaining 4232 images are divided by class-stratified random sampling in an approximately 1:2 ratio into 1411 validation images and 2821 test images. The resulting split is fixed and shared by all compared methods.

Evaluation protocol.

For the main comparisons, each compact dataset is evaluated over five downstream training runs, using the same five random seeds across methods. Test accuracy on the COVID-19 Radiography test subset with the validation-selected configurations is reported in Table 2. Table 3, Table 4 and Table 5 report the mean and sample standard deviation across five ResNet-18 runs, using each run’s highest validation accuracy. For each guidance objective and IPC budget, the COVID-19 Radiography strength with the highest mean validation accuracy is selected from Table 3. We evaluate the corresponding trained ResNet-18, ConvNet6, and ResNetAP-10 models on the COVID-19 Radiography test subset, using the same selected guidance strengths across all three architectures. For each downstream run, the checkpoint with the highest validation accuracy is used for test evaluation.

The guidance-classifier checkpoints are model010000.pt for COVID-19 Radiography, model026000.pt for OCTMNIST, and model012000.pt for BloodMNIST. These retained pretrained weights are reused across the evaluated guidance objectives and strengths. We evaluate them on clean real validation images by supplying t=0 without adding noise, using the same image preprocessing pipeline as for classifier training. For generated images, prediction–conditioning agreement is the percentage whose predicted class matches the conditioning label. An illustrative comparison between prediction–conditioning agreement and downstream utility is reported in Section 5.2.

Statistical analysis.

We additionally report 95% confidence intervals for the mean test accuracy using the sample standard deviation across five downstream runs and Student’s *t* distribution with four degrees of freedom. These intervals summarize downstream-training variability for the fixed compact datasets. Confidence intervals for all results in Table 2 are provided in Appendix A (Table A1).

Implementation details.

The COVID-19 Radiography images are resized from 299×299 to 256×256 before diffusion-model and guidance-classifier training. We use the 224×224 MedMNIST+ versions of OCTMNIST and BloodMNIST and resize them to 256×256 for the same pretraining and generation pipeline. We evaluate the compact datasets using ConvNet6, ResNet-18, and ResNetAP-10. ResNet-18 is initialized using the ResNet18_Weights.IMAGENET1K_V1 weights provided by TorchVision. These weights were pretrained on ImageNet-1K and contain no training information from the medical datasets evaluated in this study. ConvNet6 and ResNetAP-10 are initialized from scratch. ResNetAP-10 is a 10-layer residual architecture in which average-pooling operations replace strided convolutions for spatial downsampling. All experiments are conducted on an NVIDIA RTX 6000 Ada Generation GPU with 48 GB of memory. For COVID-19 Radiography, downstream classifiers are trained for 100 epochs using Adam with an initial learning rate of $10^{-3}$, zero weight decay, and a batch size of 64. StepLR multiplies the learning rate by 0.1 every 10 epochs. Inputs are 224×224 RGB images with pixel values scaled to [−1,1]. Training uses random cropping and horizontal flipping, whereas validation and test use center-crop preprocessing.

For the projected feature-space analysis, we extract 512-dimensional penultimate-layer features using a TorchVision ResNet-18 initialized with ResNet18_Weights.IMAGENET1K_V1. The final fully connected layer is replaced with an identity mapping, and feature extraction is performed in evaluation mode without gradient computation. All images are resized to 224×224 and normalized using the ImageNet mean and standard deviation. Features from real images, unguided synthetic samples, and all guided groups are concatenated before fitting a joint UMAP projection. Kernel density estimation is then applied separately to each group in the shared projected space. UMAP is configured with n_neighbors=15, min_dist=0.1, metric="cosine", and random_state=42. For each group, Gaussian kernel density estimation with Scott’s bandwidth is evaluated on a 300×300 grid covering the corresponding projected bounding box, with padding equal to one coordinate span. Dashed contours are drawn at 10% of the peak density for real samples and 30% for generated samples. Regions above the corresponding thresholds are displayed using semi-transparent fills.

Experimental settings.

To examine performance under different IPC budgets, we generate 5, 10, 20, and 50 images per class (IPC) for downstream classifier training. We compare the guided synthetic datasets with two reference settings. The first is an unguided class-conditional DDIM baseline that uses the same pretrained diffusion model without classifier-derived guidance. The second is a class-balanced Random Real subset. For each dataset and IPC budget *k*, the Random Real subset is constructed by sampling *k* images per class without replacement from the source training split. It therefore contains the same number of training images as the corresponding synthetic dataset.

For each guidance configuration, one synthetic dataset is generated using random seed 1000. The Random Real subsets and synthetic datasets are evaluated using identical image resolutions, preprocessing operations, data augmentations, downstream architectures, optimizers, learning-rate schedules, and training epochs. The downstream classifiers are trained for 100, 125, and 75 epochs on the COVID-19 Radiography, OCTMNIST, and BloodMNIST datasets, respectively.

### 5.2. Experimental Results

Table 2 reports COVID-19 Radiography test performance using guidance strengths selected on the ResNet-18 validation set and shared across architectures. With ResNet-18, all three objectives improve the mean over No Guidance at IPC =5, 10, and 20, but not at IPC =50. At IPC =5, CG raises mean accuracy from 55.20% to 66.39%. Random Real remains highest at IPC =20 and 50.

With ConvNet6, CG exceeds both reference methods at IPC =5, 10, and 20. At IPC =50, EG provides a small mean gain over No Guidance but remains below Random Real.

ResNetAP-10 shows a different pattern: all selected objectives improve on No Guidance at IPC =5 but fall below it at IPC =10. EG has the highest overall mean at IPC =20 (62.62%), and CG provides a small mean gain over No Guidance at IPC =50. Random Real leads at IPC =5 and 50. These differences indicate architecture-dependent transfer of the selected configurations.

For ResNet-18 at IPC =5, the 95% confidence intervals for the mean test accuracy are [62.61, 70.17]% for CG and [52.54, 57.86]% for No Guidance. At IPC =50, the corresponding intervals are [74.93, 80.69]% for MG and [76.15, 80.84]% for No Guidance.

Using ResNet-18 as the common downstream architecture, Table 3, Table 4 and Table 5 compare validation accuracy across guidance strengths and IPC budgets on the three datasets. These results characterize configuration sensitivity, while Table 2 separately reports the test performance of validation-selected configurations on COVID-19 Radiography.

Guidance-classifier diagnostics.

On clean real validation images evaluated at t=0, the guidance classifiers achieve accuracies of 81.36%, 94.52%, and 96.14% on COVID-19 Radiography, OCTMNIST, and BloodMNIST, respectively. On COVID-19 Radiography, class-wise accuracy ranges from 70.5% for COVID-19 images to 88.7% for normal images, indicating class-dependent prediction errors.

Prediction–conditioning agreement does not necessarily rank synthetic datasets by downstream utility. For example, at IPC =50 on BloodMNIST, CG with s=50 has agreement of 94.2%, compared with 74.0% for MG with s=3. Their corresponding downstream validation accuracies are 69.75% and 93.76%, respectively. Thus, closer agreement with the guidance classifier alone is insufficient to infer higher downstream training utility.

Downstream learning dynamics.

Figure 3 compares the downstream ResNet-18 learning curves for No Guidance and CG with s=30 on COVID-19 Radiography at IPC =5, over five runs. Training loss decreases rapidly for both configurations. No Guidance validation accuracy initially improves but declines later, whereas CG maintains a higher validation level in the later stages. The loss and validation trends are consistent with overfitting to the unguided compact dataset in this representative comparison.

### 5.3. Sensitivity to Guidance Objective and Strength

For IPC =5, 10, 20, and 50, the COVID-19 Radiography strengths selected from the ResNet-18 validation results are 30, 20, 5, and 30 for CG; 5, 3, 1, and 5 for EG; and 5, 10, 3, and 3 for MG. The across-strength standard deviation summarizes variation among the mean validation accuracies of the candidate strengths, separately from run-to-run variability.

On OCTMNIST, CG is the strongest guided objective at IPC =5, whereas EG is strongest at IPC =10, 20, and 50. At least one guided configuration exceeds both reference means at IPC =5, 10, and 50, but all evaluated guided configurations remain below both references at IPC =20. On BloodMNIST, EG is strongest at IPC =5 and MG at the larger budgets. The best guided configurations improve on No Guidance, while Random Real remains highest throughout.

Figure 4 illustrates substantial CG-strength sensitivity on OCTMNIST at IPC =5, with an across-strength standard deviation of 12.90 percentage points; accuracy varies non-monotonically across the evaluated strengths. The accompanying changes in feature-space organization are shown in Figure 5 and discussed in Section 6.3. Figure 6 and Figure 7 present the corresponding strength-sensitivity and feature-space results for BloodMNIST (13.36 percentage points), where accuracy decreases overall as strength increases. Boundary-oriented guidance can also be sensitive, as shown by OCTMNIST MG (15.20 percentage points).

## 6. Discussion

The comparison with No Guidance evaluates the contribution of classifier-derived sampling, whereas Random Real measures the gap between compact synthetic and equally sized real training sets. The framework provides additional control over synthetic-data composition; its practical value depends on the observed downstream benefit and the costs of pretraining and generation. When real images can be used directly, Random Real remains an important alternative.

### 6.1. Class Alignment Versus Boundary Exploration

CG, EG, and MG impose different preferences on the sampling trajectory. CG promotes target-class alignment, whereas EG and MG encourage different forms of predictive uncertainty. Their downstream utility may therefore depend on class structure, the composition of the compact dataset, and the downstream model’s ability to learn from the resulting examples. Prediction–conditioning agreement has different interpretations across objectives. CG directly increases the target-class log probability under the same classifier used for this diagnostic, so high agreement measures alignment with that classifier rather than independent label verification. EG and MG promote uncertainty instead of directly maximizing target-class probability. Their downstream benefit therefore need not arise from higher conditioning-label agreement, as illustrated by the BloodMNIST comparison in Section 5.2.

These considerations provide possible explanations for configuration-dependent performance, rather than a rule that predicts the optimal objective or strength in advance. We consequently use a fixed validation-based selection procedure instead of assuming a universally optimal setting. Moreover, the architecture comparisons also differ in initialization: ResNet-18 uses ImageNet-pretrained weights, whereas ConvNet6 and ResNetAP-10 are trained from scratch. Differences between these models cannot therefore be attributed solely to architecture.

### 6.2. Influence of Guidance Strength and IPC

Equation (12) shows that guidance strength scales the classifier-derived correction to the predicted noise. Increasing this strength gives the selected classifier objective greater influence over the reverse trajectory.

A plausible failure mechanism is that excessive guidance overemphasizes features favored by the guidance classifier while weakening the balance with the class-conditional generative prediction. This may reduce sample diversity, amplify classifier-specific errors, or damage visually distinguishable structures. The image and feature-space analyses can identify patterns consistent with these possibilities.

At low IPC, each generated image accounts for a larger fraction of the downstream training set, making performance more sensitive to atypical samples. Larger budgets can broaden sample coverage, but the benefit of guidance remains architecture-dependent. At IPC =50 on COVID-19 Radiography, none of the selected objectives increases the mean accuracy over No Guidance with ResNet-18, whereas EG and CG yield small mean gains with ConvNet6 and ResNetAP-10, respectively.

### 6.3. Feature-Space Behavior

The projected feature-space analysis provides a qualitative view of how CG strength changes the representation of generated samples. In the displayed OCTMNIST projection, the generated region is narrow at s=30 and expands again at s=50, indicating non-monotonic changes across the sampled configurations. BloodMNIST exhibits a more pronounced failure mode, in which high CG strength is associated with feature concentration, displacement from the real-feature centroid, and loss of distinguishable cellular structure.

These observations indicate that classifier-derived guidance can substantially alter the samples produced during reverse diffusion. However, stronger control does not necessarily produce greater downstream utility. The appropriate guidance strength depends on the visual and class structure of the dataset as well as the available IPC budget. This dependence is particularly relevant to medical dataset distillation, where compact training sets must retain subtle class-specific structures. These projections characterize similarity in the selected feature space, while modality-specific expert review would provide complementary evidence on anatomical and class-related plausibility.

### 6.4. Proposed Expert Validation Protocol

The present experiments assess the utility of synthetic images for downstream classification, rather than readiness for clinical deployment. Direct expert assessment would provide complementary evidence on whether the generated images preserve medically plausible structures and remain consistent with their conditioning labels.

We propose a blinded review with a prespecified, class-stratified sample of real images and synthetic images from No Guidance, CG, EG, and MG. The sample should include both validation-selected configurations and high-strength configurations to examine potential failure modes. Images would be presented in randomized order without identifying their source or generation method. Modality-appropriate readers would include radiologists for chest radiographs, ophthalmic imaging experts for retinal OCT, and hematology or pathology experts for blood-cell microscopy.

Readers would first assess structural plausibility, visible artifacts, and interpretability without seeing the conditioning label. Their independent class or morphology assessments could then be compared with the intended labels, allowing an uncertain or non-assessable response. For BloodMNIST, the emphasis would be on cell morphology and cell-type consistency rather than disease diagnosis. Sample size, rating criteria, and the analysis of inter-reader agreement would be specified before review.

This protocol is proposed for future validation and has not been implemented in the present study.

### 6.5. Limitations and Future Work

The evaluation is limited to public two-dimensional classification datasets. Five downstream runs are performed for each fixed generated dataset, so the reported uncertainty concerns downstream training rather than variation across independently generated datasets. ResNet-18 uses pretrained initialization, whereas ConvNet6 and ResNetAP-10 are trained from scratch; the architecture comparisons therefore combine architectural and initialization differences.

The reported COVID-19 Radiography test results use validation-selected configurations, while the OCTMNIST and BloodMNIST results characterize validation sensitivity. The COVID-19 Radiography test subset was included in the earlier combined evaluation, making the present separation a retrospective protocol revision. Evaluation on previously unused data remains future work.

Guidance-classifier diagnostics are restricted to clean-image predictions and do not directly assess error propagation along noisy sampling trajectories. Empirical privacy evaluation and the proposed expert review remain future work, together with independent generation repeats and external validation.

## 7. Conclusions

This study investigated classifier-derived guidance for diffusion-based medical dataset distillation. The proposed framework uses gradients derived from a pretrained noise-conditioned classifier to modify the reverse diffusion trajectory. Classifier guidance (CG) promotes alignment with the target class, whereas entropy and margin guidance encourage boundary-oriented sampling. The revised COVID-19 Radiography test results show that validation-selected guidance improves mean accuracy in several low-IPC settings, while its gains vary with the downstream architecture and IPC budget. The three-dataset validation analysis also identifies objective-dependent and strength-dependent operating regimes. Classifier diagnostics show that closer conditioning-label agreement does not necessarily imply higher downstream utility, and the representative downstream learning curves distinguish low training loss from sustained validation performance. The findings concern synthetic-data training utility in the evaluated settings; direct clinical validation and privacy evaluation remain future work.

## Figures and Tables

**Figure 1:**
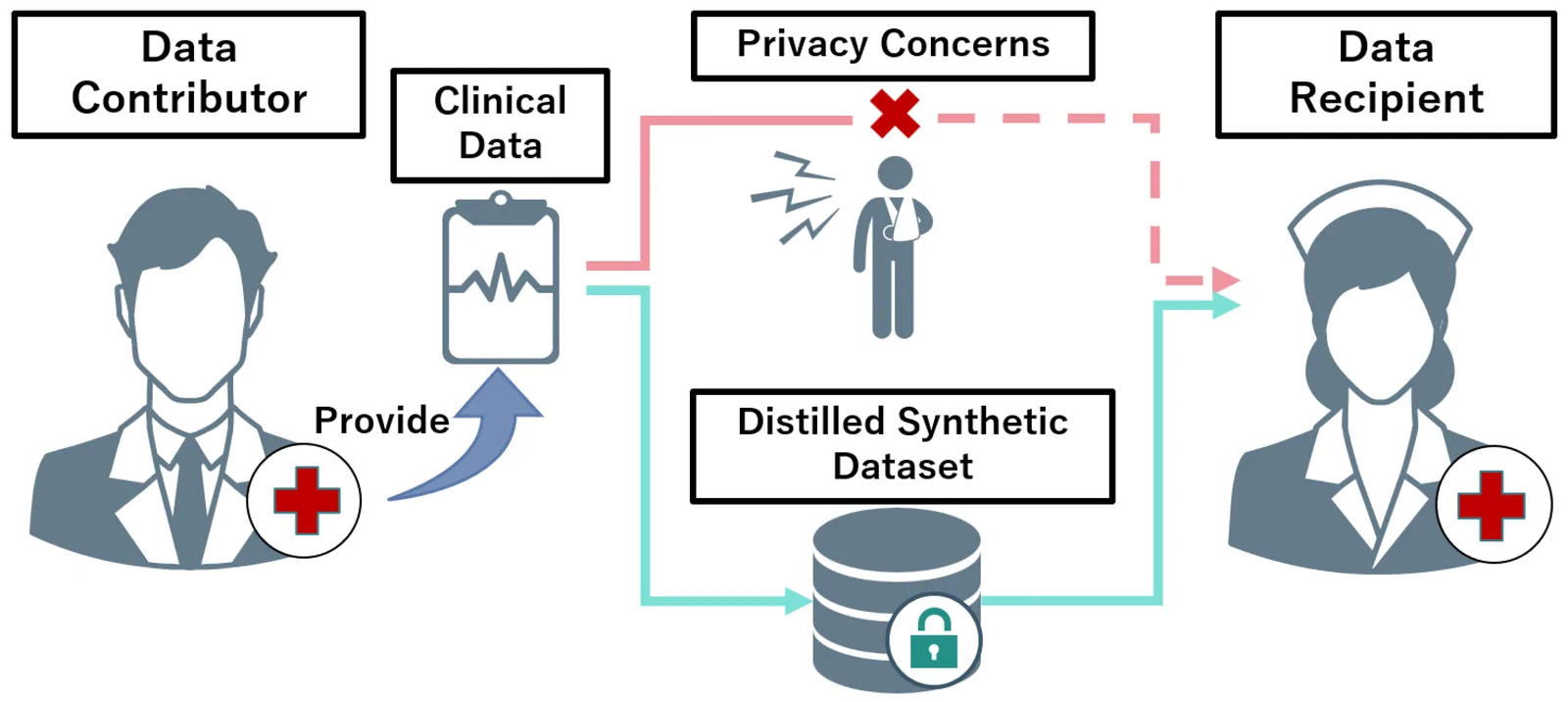
Conceptual workflow for reducing the direct exposure of source medical data through dataset distillation (DD). A source institution shares a compact synthetic dataset produced by DD rather than transferring the original medical images.

**Figure 2:**
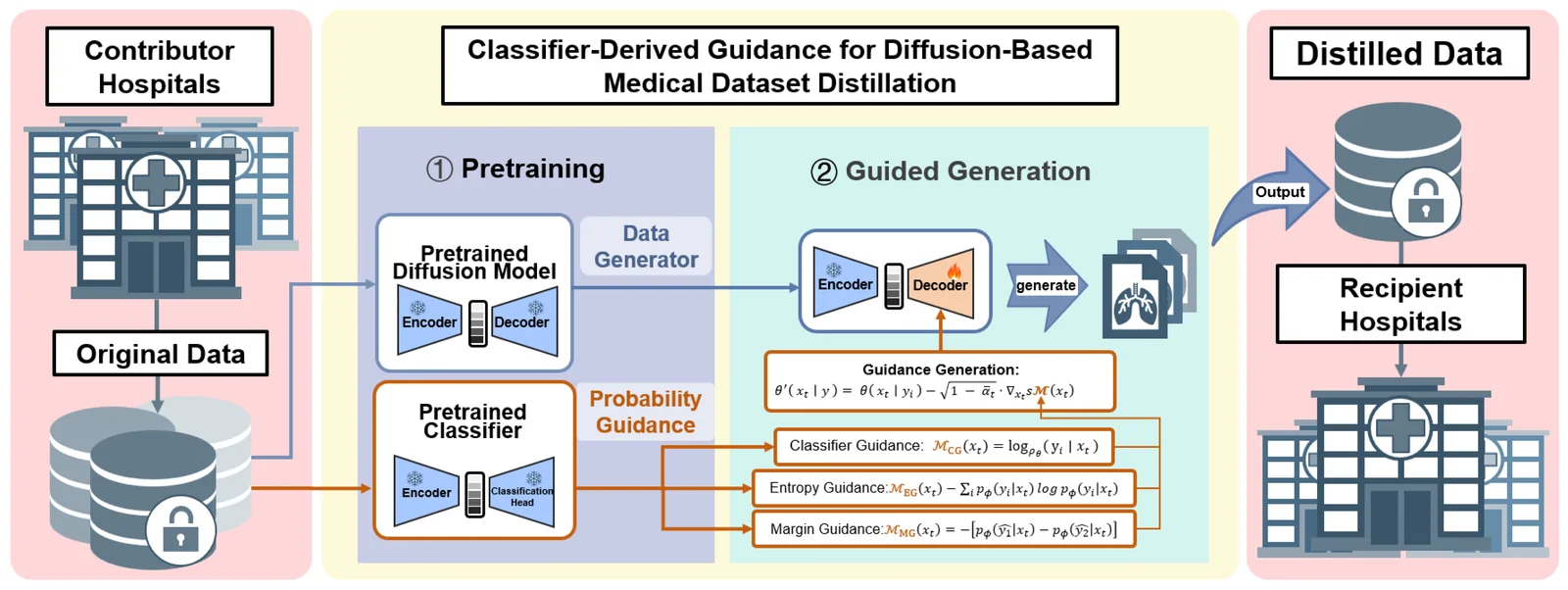
Overview of the proposed framework. A class-conditional diffusion model and a noise-conditioned guidance classifier are trained on the source training split. During DDIM sampling, the gradient of one of three classifier-derived objectives modifies the predicted noise. The resulting synthetic samples constitute a compact dataset for training a separate downstream classifier.

**Figure 3:**
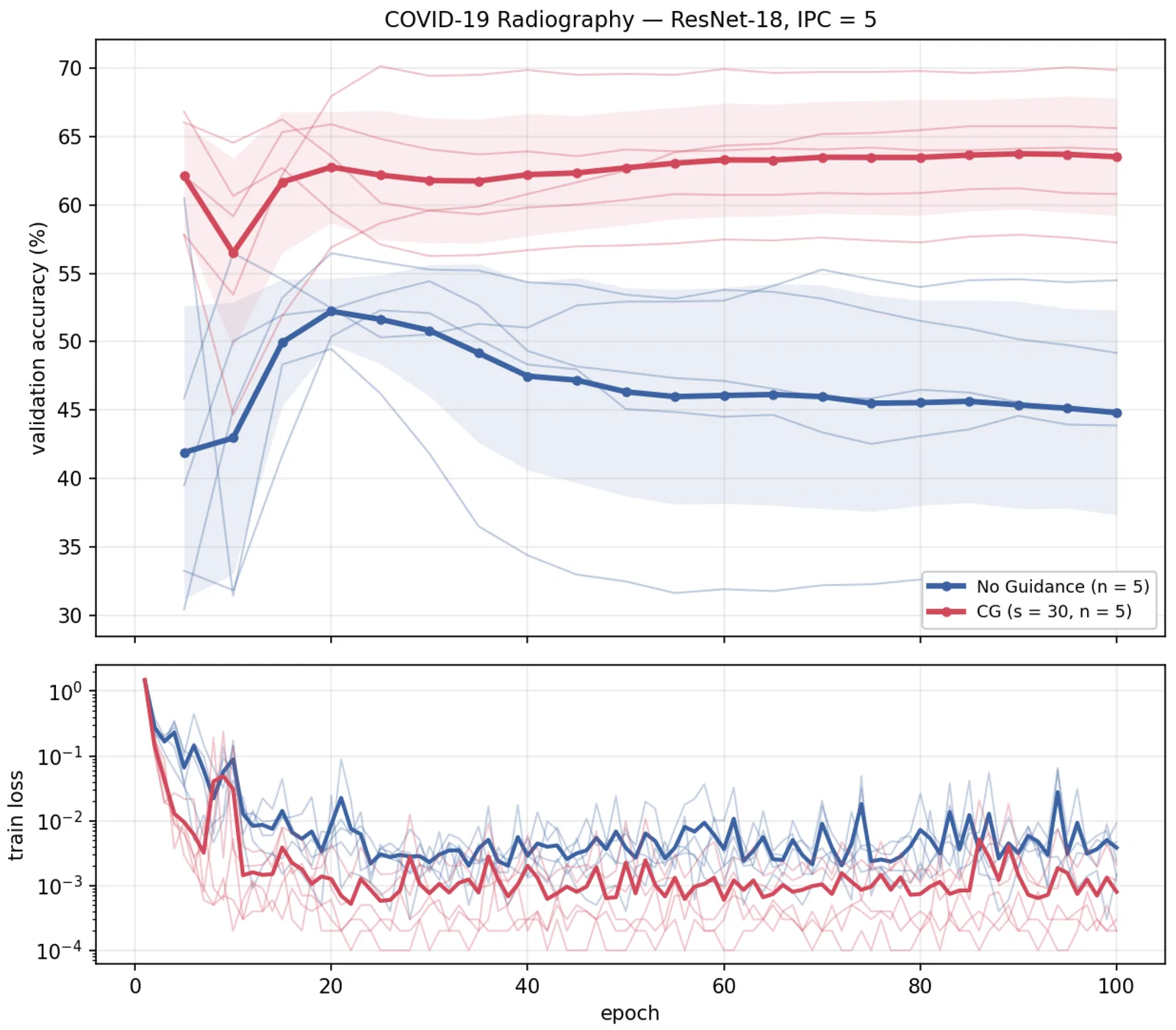
Downstream ResNet-18 learning dynamics on COVID-19 Radiography at IPC =5, comparing No Guidance and CG with s=30 over five runs. The upper panel shows validation accuracy, and the lower panel shows training loss on a logarithmic scale. Thin lines show individual runs, thick lines show their means, and the shaded bands in the upper panel indicate one standard deviation across runs. Means and standard deviations are calculated across runs at each plotted epoch.

**Figure 4:**
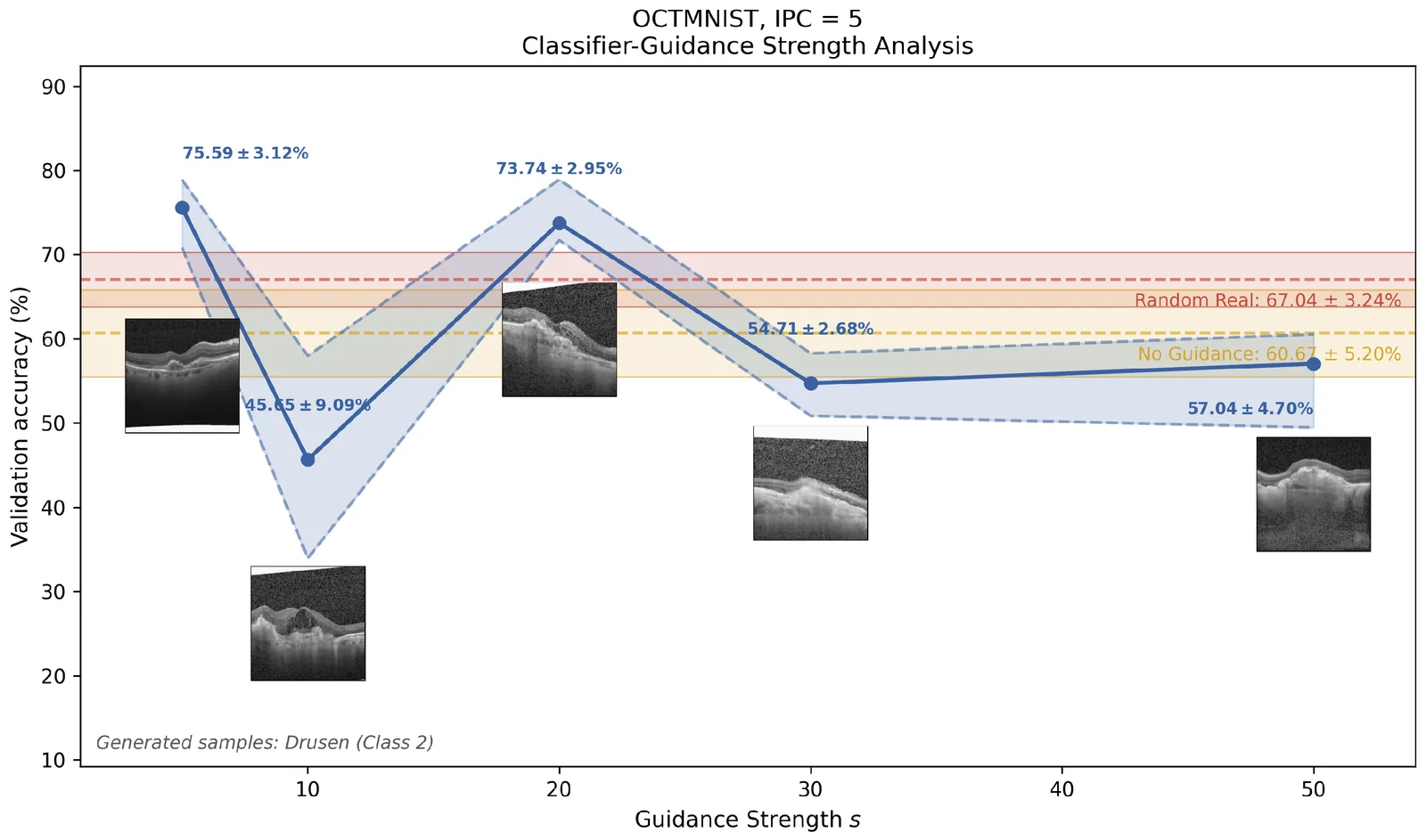
Sensitivity of OCTMNIST to CG strength at IPC =5. Curves show the mean validation accuracy and sample standard deviation over five downstream runs. Horizontal bands indicate the corresponding validation results for Random Real and No Guidance, and the insets show illustrative generated samples.

**Figure 5:**
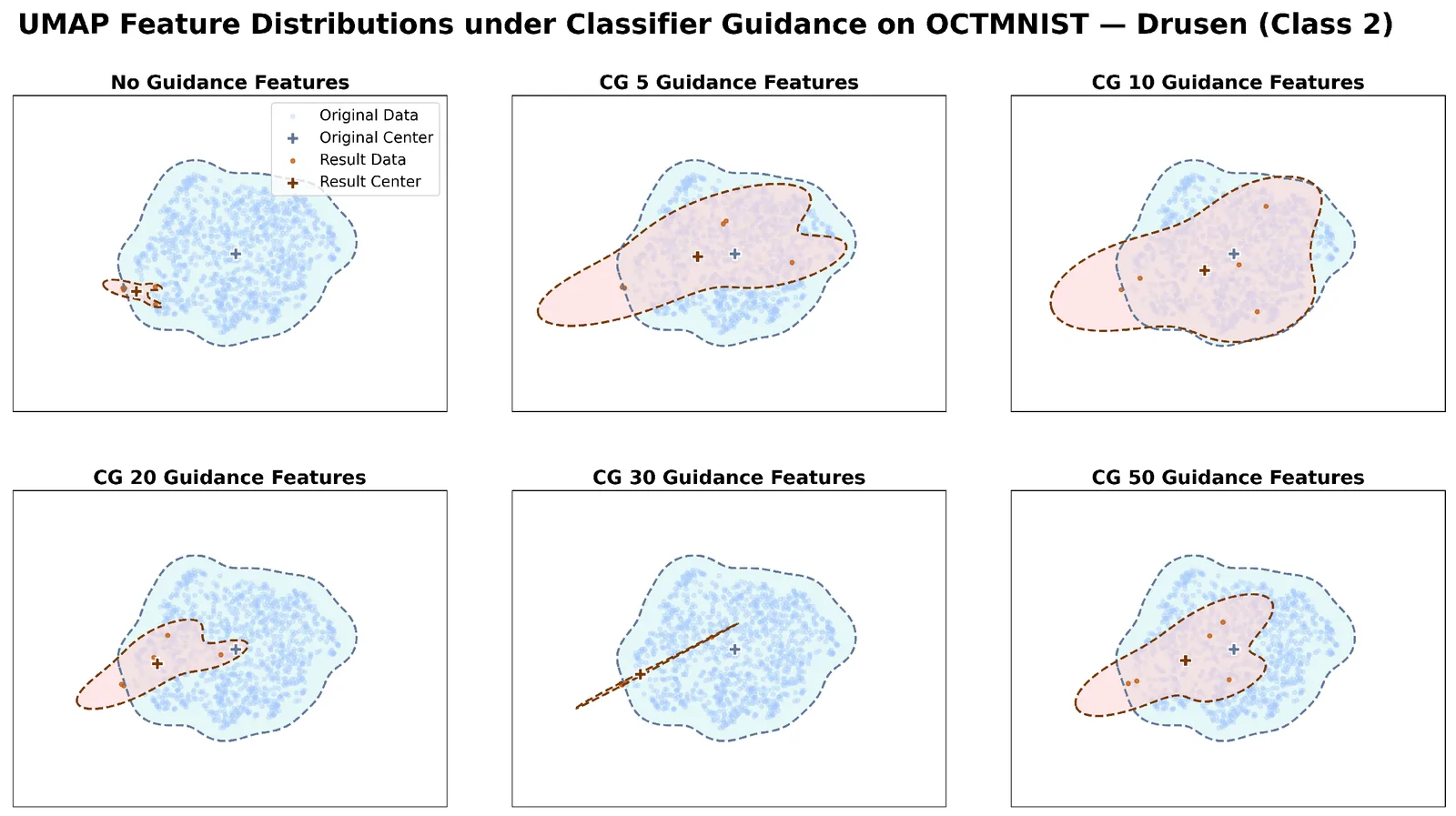
Joint UMAP projection of real and generated OCTMNIST features for the drusen class at IPC =5. Dashed contours show kernel-density level sets, and the markers indicate the corresponding centroids.

**Figure 6:**
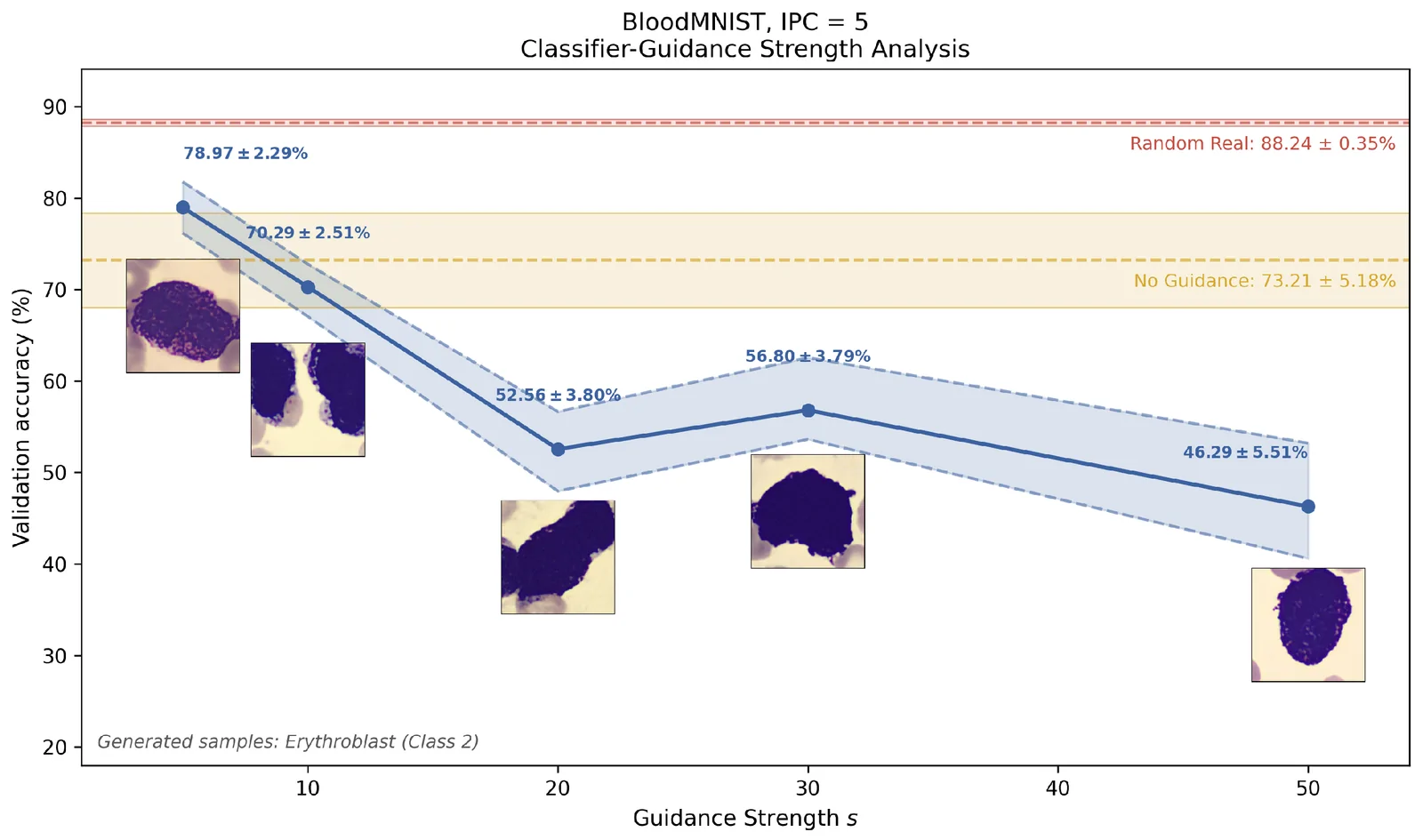
Sensitivity of BloodMNIST to CG strength at IPC =5. Curves show the mean validation accuracy and sample standard deviation over five downstream runs. Horizontal bands indicate the corresponding validation results for Random Real and No Guidance, and the insets show illustrative generated samples.

**Figure 7:**
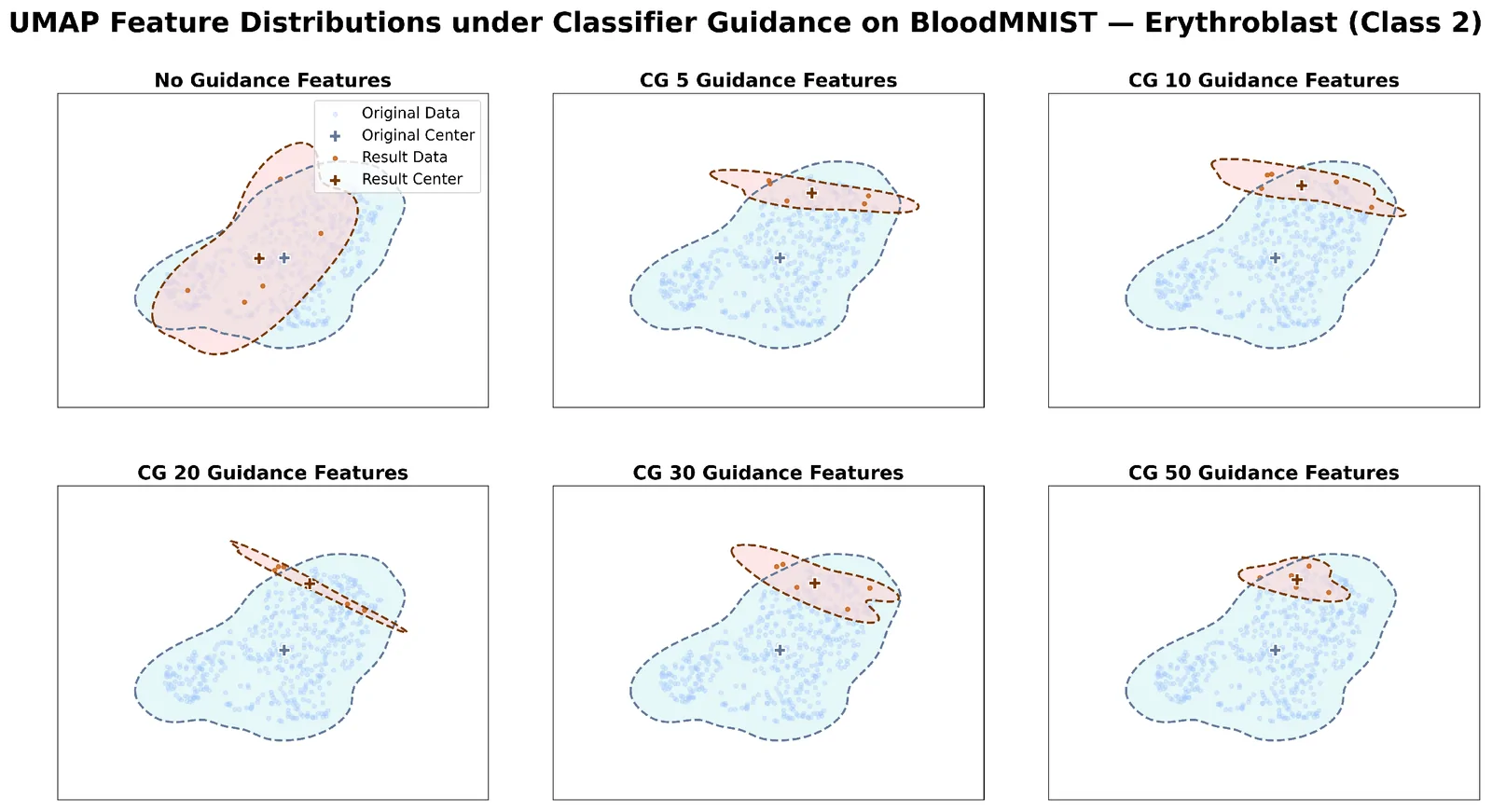
Joint UMAP projection of real and generated BloodMNIST features for the erythroblast class at IPC =5. Dashed contours show kernel-density level sets, and the markers indicate the corresponding centroids.

**Table 1:** Dataset characteristics and experimental settings.

	COVID-19 Radiography	OCTMNIST	BloodMNIST
Modality/classes	Chest X-ray (4 classes)	Retinal OCT (4 classes)	Blood cell microscopy (8 classes)
Training/validation/test	16,933/1411/2821	97,477/10,832/1000	11,959/1712/3421
Source image size	299×299	224×224	224×224
Diffusion input size	256×256	256×256	256×256
Diffusion model $\epsilon_{\theta }$	$\beta_{t}:1\times 10^{-4}\to 0.02$ (linear) T=1000 AdamW		
Diffusion training	100,000 iterations Batch size: 60 Dropout: 0.5		
Guidance classifier $p_{\phi }$	10,000 iterations	26,000 iterations	12,000 iterations
Classifier settings	Batch size: 64 Input size: 256×256 Dropout: 0.4		
GPU	NVIDIA RTX 6000 Ada Generation with 48 GB memory		
Downstream architectures	ConvNet6, ResNet-18, and ResNetAP-10	ResNet-18	ResNet-18
Epochs (LR-decay milestones)	100 [10,20,…,90]	125 [40,80,110]	75 [25,50,70]

**Table 2:** Test accuracy (%) on the COVID-19 Radiography dataset using guidance strengths selected on the ResNet-18 validation set. The same selected strengths are used across all three architectures. Results are the mean and sample standard deviation over five downstream runs. Boldface indicates the highest mean within each architecture and IPC setting.

Training Data/Guidance Method	IPC =5	IPC =10	IPC =20	IPC =50
Downstream architecture: ResNet-18				
Random Real	62.09±2.62	62.16±1.92	70.22±1.70	82.84±0.55
No Guidance (Baseline)	55.20±2.14	63.43±3.69	64.96±1.68	78.50±1.89
CG	66.39±3.04	68.06±1.49	69.65±2.04	74.19±1.38
EG	60.23±6.44	66.95±4.46	70.00±2.25	74.76±2.83
MG	56.84±4.60	67.45±3.39	66.86±3.66	77.81±2.32
Downstream architecture: ConvNet6				
Random Real	38.60±5.98	51.16±6.21	53.23±1.79	62.33±0.72
No Guidance (Baseline)	40.14±5.63	50.50±4.89	47.62±3.10	60.18±1.84
CG	56.61±4.26	57.82±1.56	58.85±0.73	59.74±1.42
EG	46.51±4.47	48.59±6.32	51.86±1.63	61.15±2.52
MG	48.81±5.77	49.93±2.36	46.28±4.77	58.55±3.35
Downstream architecture: ResNetAP-10				
Random Real	58.09±0.81	56.59±1.31	59.72±0.73	70.82±0.67
No Guidance (Baseline)	39.52±2.80	61.23±1.47	61.15±1.29	66.34±0.63
CG	48.77±1.43	54.17±1.32	61.69±1.68	67.19±1.37
EG	50.94±3.05	55.62±1.95	62.62±0.95	62.35±0.37
MG	43.87±2.83	59.20±1.70	60.96±0.69	63.20±1.08

**Table 3:** Validation accuracy (%) across guidance strengths on COVID-19 Radiography using ResNet-18. Results are the mean and sample standard deviation over five downstream runs. Boldface indicates the highest mean within each guidance objective and IPC setting. Italicized rows report the sample standard deviation of the mean accuracies across the evaluated strengths.

Method (STRENGTH)	IPC =5	IPC =10	IPC =20	IPC =50
Random Real	63.18±2.38	62.65±1.42	70.77±2.07	83.42±1.04
No Guidance (Baseline)	55.69±2.65	63.78±4.10	65.74±1.27	78.70±2.16
CG (5)	56.80±5.81	65.07±4.08	69.98±2.11	71.44±1.29
CG (10)	55.34±8.13	59.91±9.11	68.83±3.58	70.55±2.70
CG (20)	61.08±7.54	67.90±1.29	67.74±3.46	71.27±3.47
CG (30)	65.90±3.16	65.44±5.15	68.93±2.08	74.70±1.54
CG (50)	63.78±2.46	57.28±3.76	66.17±1.93	66.17±2.11
*CG Across-strength SD*	*4.49*	*4.37*	*1.44*	*3.06*
EG (1.0)	45.87±7.67	63.26±1.78	70.28±2.13	70.59±3.42
EG (3.0)	52.33±9.34	66.87±4.84	63.64±5.75	69.24±4.73
EG (5.0)	59.48±6.48	54.56±4.86	67.85±2.83	74.73±4.00
EG (10.0)	43.56±9.84	39.36±3.27	68.90±2.14	74.44±1.76
*EG Across-strength SD*	*7.15*	*12.25*	*2.86*	*2.75*
MG (1.0)	52.01±10.22	62.06±2.48	66.95±7.33	75.65±1.12
MG (3.0)	51.17±3.49	59.70±3.79	67.33±3.36	78.48±2.91
MG (5.0)	57.55±4.40	66.17±4.94	63.78±6.39	72.18±3.77
MG (10.0)	45.06±6.87	66.75±4.01	62.47±6.42	77.94±1.51
*MG Across-strength SD*	*5.11*	*3.37*	*2.38*	*2.86*

**Table 4:** Validation accuracy (%) across guidance strengths on OCTMNIST using ResNet-18. Results are the mean and sample standard deviation over five downstream runs. Boldface indicates the highest mean within each guidance objective and IPC setting. Italicized rows report the sample standard deviation of the mean accuracies across the evaluated strengths.

Method (Strength)	IPC =5	IPC =10	IPC =20	IPC =50
Random Real	67.04±3.24	74.66±4.33	82.66±2.17	86.78±1.36
No Guidance (Baseline)	60.67±5.20	73.09±3.03	80.68±2.78	85.01±3.25
CG (5)	75.59±3.12	71.90±7.49	78.78±4.94	83.22±2.99
CG (10)	45.65±9.09	67.36±8.26	77.11±3.25	82.13±3.25
CG (20)	73.74±2.95	71.53±3.46	78.96±3.13	74.52±4.53
CG (30)	54.71±2.68	62.10±9.70	74.85±6.40	72.96±3.90
CG (50)	57.04±4.70	67.15±9.27	70.11±3.25	74.76±3.04
*CG Across-strength SD*	*12.90*	*3.99*	*3.67*	*4.77*
EG (1.0)	43.21±7.76	65.71±5.81	72.71±8.40	87.41±1.44
EG (3.0)	60.79±9.00	56.87±10.24	77.90±1.94	82.76±2.81
EG (5.0)	51.49±3.97	77.12±1.70	66.91±9.74	73.47±9.70
EG (10.0)	51.80±5.26	69.19±8.05	79.45±5.25	84.29±3.54
*EG Across-strength SD*	*7.18*	*8.39*	*5.67*	*6.00*
MG (1.0)	55.81±5.54	71.26±11.81	66.15±6.20	74.67±10.30
MG (3.0)	30.34±5.46	65.71±7.44	70.04±8.98	85.72±1.87
MG (5.0)	57.41±3.62	55.80±14.88	73.88±5.28	85.71±1.38
MG (10.0)	65.43±11.49	69.30±7.41	78.43±1.75	83.25±3.47
*MG Across-strength SD*	*15.20*	*6.87*	*5.26*	*5.24*

**Table 5:** Validation accuracy (%) across guidance strengths on BloodMNIST using ResNet-18. Results are the mean and sample standard deviation over five downstream runs. Boldface indicates the highest mean within each guidance objective and IPC setting. Italicized rows report the sample standard deviation of the mean accuracies across the evaluated strengths.

Method (Strength)	IPC =5	IPC =10	IPC =20	IPC =50
Random Real	88.24±0.35	90.26±1.97	94.66±0.28	97.02±0.36
No Guidance (Baseline)	73.21±5.18	83.53±4.87	86.16±3.19	91.62±2.44
CG (5)	78.97±2.29	78.31±3.16	82.99±2.38	87.08±0.63
CG (10)	70.29±2.51	76.58±2.84	80.54±1.32	85.91±1.09
CG (20)	52.56±3.79	67.98±6.24	72.63±5.31	77.91±2.35
CG (30)	56.80±3.80	69.65±5.47	68.35±7.37	76.86±2.77
CG (50)	46.29±5.51	52.01±5.65	61.06±6.90	69.75±3.11
*CG Across-strength SD*	*13.36*	*10.42*	*8.96*	*7.13*
EG (1.0)	75.97±6.73	79.45±2.88	87.91±3.01	92.23±0.68
EG (3.0)	70.36±4.55	78.76±2.84	86.66±1.74	92.92±0.76
EG (5.0)	81.73±1.60	78.28±1.89	86.55±1.80	92.66±0.56
EG (10.0)	71.97±7.43	71.71±2.53	87.55±2.01	92.10±1.54
*EG Across-strength SD*	*5.06*	*3.59*	*0.67*	*0.38*
MG (1.0)	77.80±2.59	82.55±2.87	86.69±1.95	91.61±0.75
MG (3.0)	73.62±5.91	83.62±2.65	90.63±0.92	93.76±0.75
MG (5.0)	71.33±4.38	85.12±2.08	88.71±2.17	91.55±1.01
MG (10.0)	67.23±7.68	80.23±2.90	89.91±0.73	91.39±0.75
*MG Across-strength SD*	*4.42*	*2.06*	*1.72*	*1.13*

## Data Availability

The source datasets used in this study are publicly available from the MedMNIST/MedMNIST+ repository and the COVID-19 Radiography Database at the sources cited in this article. The generated compact datasets, data-split files, training configurations, and evaluation scripts are available from the corresponding author upon reasonable request.

## Appendix A. Confidence Intervals for Table 2

**Table A1:** The 95% confidence intervals for the mean test accuracies reported in Table 2 on COVID-19 Radiography. The configurations and five downstream runs are the same as those used in Table 2. All values are percentages.

Method	IPC =5	IPC =10	IPC =20	IPC =50
ResNet-18				
Random Real	[58.84,65.34]	[59.78,64.55]	[68.11,72.32]	[82.16,83.51]
No Guidance	[52.54,57.86]	[58.85,68.02]	[62.87,67.05]	[76.15,80.84]
CG	[62.61,70.17]	[66.21,69.91]	[67.12,72.18]	[72.48,75.90]
EG	[52.23,68.23]	[61.40,72.49]	[67.21,72.79]	[71.25,78.27]
MG	[51.12,62.55]	[63.24,71.66]	[62.32,71.41]	[74.93,80.69]
ConvNet6				
Random Real	[31.18,46.03]	[43.45,58.86]	[51.00,55.45]	[61.43,63.23]
No Guidance	[33.16,47.13]	[44.43,56.57]	[43.77,51.47]	[57.90,62.47]
CG	[51.32,61.90]	[55.88,59.76]	[57.95,59.76]	[57.97,61.50]
EG	[40.96,52.06]	[40.74,56.44]	[49.84,53.88]	[58.02,64.28]
MG	[41.65,55.97]	[47.01,52.86]	[40.36,52.20]	[54.39,62.72]
ResNetAP-10				
Random Real	[57.09,59.10]	[54.96,58.22]	[58.81,60.63]	[69.98,71.66]
No Guidance	[36.05,43.00]	[59.40,63.06]	[59.55,62.74]	[65.55,67.13]
CG	[46.99,50.55]	[52.53,55.80]	[59.60,63.78]	[65.49,68.89]
EG	[47.15,54.73]	[53.19,58.04]	[61.43,63.80]	[61.89,62.80]
MG	[40.36,47.38]	[57.09,61.31]	[60.10,61.82]	[61.86,64.55]
